# Supplementary material for: Periplaneta americana extract ameliorates recurrent oral ulcers in rats by enhancing the intestinal epithelial barrier and regulating gut microbiota
Source: PLoS One. 2026 Jan 23;21(1):e0340453. doi: 10.1371/journal.pone.0340453 (PMC12829797; doi:10.1371/journal.pone.0340453)
Supplement: S1 File — (DOCX) [file pone.0340453.s001.docx]

**Graphical Abstract**


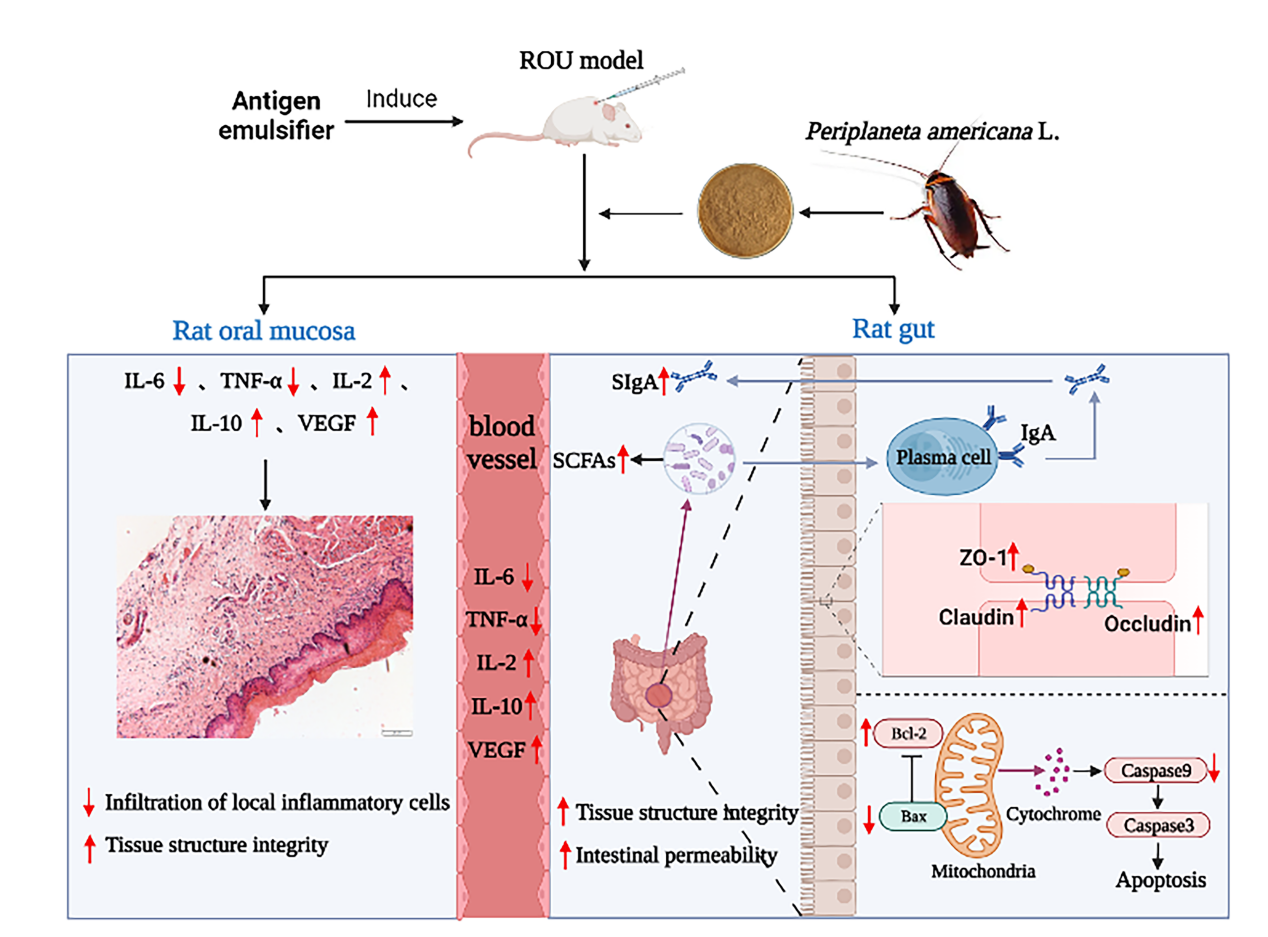


**Fig.** Graphical abstract of amelioratory effects of *Periplaneta americana* extract on rats with recurrent oral ulcers by enhancing the intestinal epithelial barrier and regulating gut microbiota.
