# Supplementary material for: Periplaneta americana extract ameliorates recurrent oral ulcers in rats by enhancing the intestinal epithelial barrier and regulating gut microbiota
Source: PLoS One. 2026 Jan 23;21(1):e0340453. doi: 10.1371/journal.pone.0340453 (PMC12829797; doi:10.1371/journal.pone.0340453)
Supplement: Attachment — Submitted filename: reviwer comments (7).docx [file pone.0340453.s003.docx]

**Reviewer Comments on Manuscript PONE-D-25-27151**

**Title:** Periplaneta americana extract ameliorates recurrent oral ulcers in rats by enhancing the intestinal epithelial barrier and regulating gut microbiota

**Summary**

The manuscript presents a well-structured study exploring the therapeutic potential of *Periplaneta americana* extract (PAD) in a rat model of recurrent oral ulcers (ROUs). The work combines histological, molecular, and microbiota analyses to support its conclusions. The topic is novel and relevant, with potential implications for oral disease treatment and gut–oral microbiota research. However, there are notable concerns about experimental design, interpretation, and presentation.

Novelty: The study connects PAD treatment with both intestinal barrier function and microbiota regulation, which is a relatively unexplored therapeutic pathway in oral ulcer management.

Methodological breadth: The authors employed multiple approaches (histopathology, ELISA, RT-qPCR, WB, immunohistochemistry, 16S rRNA sequencing, GC-MS), strengthening the credibility of findings.

Clear results: Data show consistent improvements in ulcer healing, inflammatory cytokine modulation, SCFA restoration, and tight junction protein expression.

Potential clinical relevance: Findings may encourage translational research into PAD-derived products for human use.

**Major Comments**

Model justification: The antigen emulsifier-induced ROU model is not standard. The authors should provide stronger justification that this model reflects the pathophysiology of human recurrent aphthous ulcers, and discuss its limitations.

The conclusion should be tempered to emphasize association rather than causality.

Overstated conclusions: The manuscript at times suggests PAD is an “effective treatment” for ROUs. Given that the data are preclinical (rat model only), the discussion should highlight translational limitations and avoid clinical overstatements.

Clarity and redundancy: The Introduction and Discussion are long and at times repetitive. Streamlining would improve readability.

**Minor Comments**

- Figures and legends should be checked for clarity (some are difficult to interpret in the PDF).
- Abbreviations (PAD, ROU, SCFA, GM) should be redefined in Results for reader convenience.
- Ethical approval is mentioned, but animal welfare descriptions could be expanded (analgesia, humane endpoints).
- The limitation section briefly mentions oral flora, but further elaboration is needed (e.g., small sample size, lack of female animals).
- References: Some claims in Introduction and Discussion would benefit from more recent citations.
- Grammar and syntax require polishing for international readability.

**Recommendation: acceptance with minor revision**

The study provides interesting and potentially impactful findings, but substantial revisions are required before acceptance. Specifically, the authors should (1) justify the animal model, (2) moderate mechanistic and clinical claims, (3) strengthen the microbiota analysis, and (4) improve clarity and English expression.
