# Supplementary material for: Periplaneta americana extract ameliorates recurrent oral ulcers in rats by enhancing the intestinal epithelial barrier and regulating gut microbiota
Source: PLoS One. 2026 Jan 23;21(1):e0340453. doi: 10.1371/journal.pone.0340453 (PMC12829797; doi:10.1371/journal.pone.0340453)
Supplement: Attachment — Submitted filename: Response to Reviewers.docx [file pone.0340453.s006.docx]

**Response to Reviewers**

Manuscript Number: **PONE-D-25-27151**

***Periplaneta americana* extract ameliorates recurrent oral ulcers in rats by enhancing the intestinal epithelial barrier and regulating gut microbiota**

Dear Reviewers,

Thank you very much for your valuable, detailed, and professional comments and queries about the scientific content in this revised manuscript, which gave us clear direction and scientific guidance during the revision process. All the authors and I have made detailed, targeted, and comprehensive revisions and answers to the questions raised by the reviewers together in the relevant manuscript. The revised content is marked in blue font within the manuscript. The page numbers and line numbers mentioned correspond to the annotations in the “Revised Manuscript with Track Changes” file. The responses to the suggestions made by the editors and reviewers are as follows:

Editor:

1. About the question “Please make sure that all relevant research efforts of other groups are adequately cited in the manuscript—both in favor/support and against the current research.”

**Reply:** Thank you for your valuable suggestions—this requirement is crucial for enhancing the academic rigor and completeness of the research. Firstly, we have systematically searched recent research literature in the relevant field, comprehensively sorted out academic achievements that support or differ from the conclusions of this study, and ensured that no important related research is omitted. Secondly, we will supplement and standardize citations in relevant sections such as the introduction and discussion: we will respond to studies supporting this research, objectively present studies with different viewpoints, and analyze the possible reasons for the discrepancies in combination with the research design and results of this study. Finally, all supplementary citations will strictly follow the reference format requirements of the target journal to ensure that the citations are standardized and accurate. We will earnestly implement the above work, striving to make the manuscript more comprehensive in academic perspective and more sufficient in argumentation. Thank you again for your rigorous review and careful guidance.

2. About the question “Please ensure that the methodology is detailed and all information for reagents has been included.”

**Reply:** Thank you for your rigorous suggestions. Detailed and comprehensive research methods, along with complete reagent information, are the core foundation for ensuring the reproducibility and scientificity of the research. We have conducted a rigorous review of every item to ensure no omissions or errors, thereby comprehensively enhancing the standardization and transparency of the research methods. Thank you for your careful review, which will provide an important guarantee for the credibility of the research results.

3. About the question “Please ensure scale bars, labels, resolution of images are appropriate and adherent to Journal guidelines.”

**Reply:** Thank you for your careful reminder. We have conducted a thorough check of the scale bar settings for all experimental images, ensuring that the scale bar calibrations are consistent with the image magnification and clearly labeled at an appropriate position on each image. We have standardized the image labeling information, unified the font, font size, and labeling format, and ensured that key information such as group designations and indicators is clear and unambiguous. In accordance with the journal’s image resolution requirements, we have inspected and optimized all images. If any images fail to meet the resolution standard, they will be re-exported or reprocessed to ensure compliance with the journal’s publication criteria.

4. About the question “Please ensure that statistical analyses are rigorously performed for all data. Please also ensure that data shown in figures are representative of at least three independent experiments performed in replicates.”

**Reply:** Thank you for your key suggestions regarding the rigor of statistical data analysis and experimental reproducibility, which are of paramount importance for ensuring the reliability and scientific validity of the research results. During the comprehensive review of the entire manuscript, we have systematically implemented the relevant requirements and conducted rigorous verification, effectively ensuring the transparency of the research. Thank you again for your rigorous review, which has further enhanced the scientific rigor and standardization of the manuscript.

5. About the question “Please avoid/remove any overstatements in the manuscript. Please ensure that the statements are based on results obtained.”

**Reply:** Thank you for your crucial reminder. We have conducted multiple verifications to ensure that all statements in the manuscript are fully based on the experimental results, without any exaggerated or false content. Thank you again for your professional review, which will help us more accurately present the research value and uphold the objectivity and rigor of academic research.

**Reviewer #1:**

1. About the question “The author should carry out experiments to understand the chemo profile of Periplaneta americana extract. Without knowing what is contains, its not justified to consider it as therapeutic agent..”

**Reply:** Thank you very much for your interest in PAD bioactive components and standardization issues. Your feedback provides valuable guidance for us to more clearly convey the research background and value. We have supplemented the discussion section with 40 PAD (*Periplaneta americana* extract) components previously identified by the research team through UPLC-Q-TOF-MS analysis. These include 7 amino acids, 7 alkaloids, 6 fatty acids, 5 nucleosides, 4 peptides, 3 sugars, and 8 other compounds (Page 23, lines 510-512). Additionally, formulations primarily composed of PAD (Kangfuxin Liquid) have been applied in the treatment of oral mucosal epithelial damage caused by radiation therapy for tumors. The core objective of this study is to address the current situation where its clinical efficacy is well-established, but its mechanism of action remains unclear. We aim to delve into the specific pathways through which it improves oral ulcers by regulating the GM, thereby filling the gap in mechanism research. Thank you once again for your meticulous review and professional feedback, which has enabled the manuscript to more fully convey its value to PAD-related research.

2. About the question “Dose dependent toxicity studies are required.”

**Reply:** Thank you very much for pointing out the omissions in our manuscript. At present, preparations based on PAD (including Kangfuxin Liquid and toothpaste) have been widely used in clinical treatment and daily care scenarios. In addition, we previously conducted acute toxicity tests on this extract, and the results showed that the maximum tolerated dose (MTD) of PAD in mice exceeds 40 g/kg, with no toxicity observed and a good safety profile. Relevant experimental results are shown in the figure below:

3. About the question “The precise molecular markers related with recurrent oral ulcers should be carried out using a model cell line as well in in vivo model study, simply apoptosis related markers is not enough.”

**Reply:** We sincerely appreciate your insightful and constructive key recommendations. We fully acknowledge the limitations of relying solely on apoptosis-related markers in existing research, and your perspective provides a clear direction for our subsequent studies. Moving forward, we may design plans to further deepen the investigation of relevant molecular mechanisms through cellular experiments, thereby addressing the shortcomings of current research.

4. About the question “There is need to select a positive control for comparison purpose.”

**Reply:** Thank you very much for your comment. The positive control drug in this study is levamisole hydrochloride (LM) tablets (Page 8, lines 158 and 174), and we have explained the rationale for selecting this drug as the positive control in the Discussion section (Page 24, lines 531-541).

5. About the question “The MS has been written in very poor manner.”

**Reply:** We sincerely appreciate your frank feedback and rigorous review. We have comprehensively sorted out the logical structure, academic expressions, and detailed norms of the manuscript, focusing on optimizing the coherence, accuracy, and fluency of the content. We have rectified possible omissions and inappropriate points one by one, striving to significantly improve the manuscript quality and live up to your review efforts. Thank you again for your critical comments and corrections, which are of vital importance for us to improve the research results and enhance the manuscript quality.

**Reviewer #2:**

About the question “This research is exceptionally well-designed and meticulously executed. It presents a highly engaging and valuable contribution to the therapeutic field of ulcer, employing a wide range of advanced techniques to robustly support and validate the proposed hypothesis.”

**Reply:** We sincerely appreciate your high recognition and valuable feedback on this research. Your affirmation not only validates the value of this research within the field, but also strengthens our determination to continue delving deeply into this area and promoting the transformation of research results into practical applications. It also injects confidence into our future exploration of cross-technology integration applications. This undoubtedly will become an important driving force for us to further improve the paper and deepen related research in the future.

**Reviewer #3:**

About the question “The manuscript presents a well-designed study on the therapeutic effects of *Periplaneta americana* extract (PAD) in a rat model of recurrent oral ulcers. It provides valuable insights into PAD’s multi-target actions through the gut-oral-immune axis. The findings support PAD's potential as a natural therapeutic agent, especially in traditional Chinese medicine. The study contributes meaningfully to the field of natural compound-based therapy and mucosal immunology.”

**Reply:** We sincerely thank you for taking the time to carefully and deeply review this research and for your high praise and precise evaluation. Your feedback not only shows our deep understanding of the research content, but also greatly encourages our team to receive professional recognition for our previous efforts. We will carry forward your recognition and continue to delve deeply into the fields of natural drug therapy and mucosal immunology, striving to produce more valuable research results.

**Reviewer #4:**

**Abstract**

1. About the question “Some meanings of the acronyms were not expressed in this section, such as SCFAs, RT-qPCR, and WB. They should be given in their full form when they were first used in the text to increase clarity.”

**Reply:** Thank you very much for your valuable suggestions. We have carefully checked the usage of all abbreviations in the manuscript and supplemented the full names of abbreviations such as SCFAs, RT-qPCR, and WB (Page 2, lines 25-34). We ensured that all abbreviations followed the rule of “the complete form is indicated for the first occurrence”, providing clearer guidance for readers to understand the research content.

2. About the question “I would prefer a structured abstract rather than a uniform text.”

**Reply:** We sincerely appreciate your valuable suggestions, which have provided crucial guidance for enhancing the manuscript's quality. We sincerely appreciate your valuable suggestions, which have provided crucial guidance for enhancing the manuscript's quality. Based on your recommendations, we have revised the original narrative abstract into a structured abstract. By clearly delineating the research background and objectives, methods, results, and conclusions, we have organized key information more systematically to enable readers to quickly grasp the core content of the study (Page 2, lines 15, 21, 24, 31, and 38).

3. About the question “Except for these, this section clearly summarizes the objective, methods, and results of the study.”

**Reply:** Thank you very much for your recognition of this section. During the writing process, we have always strived to present the core information of the research in a more intuitive way. At the same time, we have also taken note of the other areas for improvement you mentioned earlier and have made the necessary revisions. While maintaining the clear and concise presentation of this section, we have further refined the details to ensure that the overall content is more rigorous and comprehensive.

**Introduction**

1. About the question “While the graphical abstract well summarizes the study, the term located in the figure explanation “ameliorates” should be changed to “ameliorating” or “amelioratory” in the revised version.”

**Reply:** Thank you very much for your recognition of the Graphical Abstract and for your valuable suggestions regarding the revision of terminology. We have changed “ameliorates” in the legend description to “amelioratory” to ensure that the term usage and grammatical logic are more consistent (Page 3, line 48), while maintaining the clarity of the legend description in Graphical Abstract.

2. About the question “ ‘While ROU attacks are localized and typically resolve on their own, the intense burning pain, swollen lymph nodes, general malaise, and other symptoms that come with the onset of the condition can significantly impact patients' mental well-being and quality of life.’ Although I agree with the fact that the symptoms can decrease the quality of life, as a patient with ROUs, I found the term "impacting the mental health significantly" to be extravagant. For me, this disease is a mild disease without a severe, life-threatening symptoms and complications.”

**Reply:** Thank you very much for your valuable suggestions. Your opinions will enable us to improve the accuracy of our expressions and better align with the actual feelings of the patients. We have revised the original phrasing, replacing “While ROU attacks are localized and typically resolve on their own, the intense burning pain, swollen lymph nodes, general malaise, and other symptoms that come with the onset of the condition can significantly impact patients' mental well-being and quality of life” with “While ROU attacks are localized and typically resolve on their own, the intense burning pain, swollen lymph nodes, general malaise, and other symptoms that accompany these attacks still interfere to some extent with the patient's daily life and have an impact on their quality of life” (Page 4, lines 56-60). Thank you once again for your professional feedback from a patient's perspective. This helps our content better align with clinical practice and the authentic patient experience.

3. About the question “ ‘Currently, ROU is clinically treated with Western medicine, and the commonly used drugs are glucocorticoids, thalidomide, and colchicine, but they have adverse effects such as teratogenicity, dizziness, and gastrointestinal adverse reactions’. In this sentence, the authors presented the ROUs similar to the clinical symptoms and treatment of Behçet's Disease. However, isolated ROUs are free from multisystemic involvement and rheumatological clinical progress. Therefore, to avoid confusion in readers, the authors should clearly define and illustrate the clinical picture of the ROUs.”

**Reply:** Thank you very much for your valuable suggestions. We have clearly outlined the clinical symptoms of ROU in the introduction section to ensure that the relevant background information is comprehensive and well-defined (Page 4, lines 56-60).

4. About the question “Except for these, the introduction section clearly explains the objective of the study and pathophysiological interactions between the ROUs, intestinal barrier, and Chinese traditional therapies.”

**Reply:** We sincerely appreciate your recognition of the Introduction section of our manuscript. This recognition not only serves as encouragement for our writing efforts, but also provides us with crucial confidence to further refine the manuscript. We always maintain a rigorous attitude to polish the manuscript.

**Method**

1. About the question “ ‘Complete Freund’s adjuvant, butyric, propionic, butyric, and 101 pentanoic were purchased from Sigma-Aldrich (St. Louis, MO, USA).’. ‘Butyric’ is repeated in this sentence.”

**Reply:** Thank you very much for your meticulous and rigorous review. We have verified and corrected the manuscript, changing the first “Butyric” to “acetic” (Page 6, line 111). Moreover, after the correction, we have ensured that the names of the reagents used in this study are accurate, without any repetition or redundancy. We once again appreciate your professional feedback. Your meticulous assistance enabled us to promptly correct the expression errors and further ensured the rigor of the manuscript.

2. About the question “"UPLC-Q-TOF-MS (Agilent, USA). Microscope (Olympus, Japan). GC-MS system 118 (Agilent, USA). QuantiFluor™-ST blue fluorescence quantitative system (Promega, 119 Madison, WI, USA). Illumina MiSeq platform (Shanghai Yuan Shen Biomedical 120 Technology Co. Ltd., Shanghai, China). Ultramicro nucleic acid and protein analyzer 121 (Thermo Fisher Scientific, Waltham, MA, USA). CFX96 Real-Time PCR Detection 122 System (Bio-Rad Laboratories, Hercules, CA, USA). Image-Pro Plus 6 software (Media 123 Cybernetics, Inc., Rockville, MD, USA). SPSS 26.0 statistical software (SPSS Inc., 124 Chicago, IL, USA)." There is no meaningful sentence in this part except brand names.”

**Reply:** Thank you for pointing out the issue with the presentation of information in the “2.1 Materials and Reagents” section. We have removed the instrument and software information that solely listed brands from this section and incorporated these details into the corresponding experimental steps within the “2. Material and methods” section (such as page 6, line 127, page 7, lines 128-134, page 10, lines 201, 213, and page 11, lines 232, 234-235, 247-248).

3. About the question “Except for these, the methods section is very detailed and explains all of the steps performed during the study.”

**Reply:** Thank you for your thorough endorsement of the methods section of this study, which is crucial for ensuring the reproducibility of the research. Throughout the methods section, we adhered to the principles of clarity, completeness, and reproducibility, striving to provide detailed explanations of every critical step in the research process—from sample preparation and experimental procedures to data analysis. Your affirmation not only recognizes our work but also further strengthens our resolve to continuously refine methodological descriptions and ensure research rigor in subsequent studies.

**Results:**

About the question “The authors supported their results with very detailed figures, illustrations, and demonstrations.”

**Reply:** Thank you for recognizing the supporting materials for this research. This approach is precisely the key design principle we adhere to to enhance the readability and persuasiveness of our study. We consistently emphasize enhancing the supporting evidence of our findings through visualization: ensuring key results are quantifiable and traceable while helping readers quickly grasp the research framework, ultimately making the supporting evidence for our research outcomes more robust and intuitive.

**Discussion:**

About the question “The discussion section widely explains the results of the study. Numerous references and comparisons with similar studies in the literature are available.”

**Reply:** We sincerely appreciate your acknowledgment of the “Discussion” section of this manuscript. In drafting the “Discussion” section, we comprehensively review the core findings of the study. We not only present the data conclusions but also delve into the scientific implications and underlying mechanisms behind the results, striving to articulate a logically coherent narrative. Additionally, we systematically retrieved and referenced relevant literature within the field. By comparing our findings with those of similar studies, we identified consistent results and innovative differences in this research. Simultaneously, we objectively analyzed potential reasons for these discrepancies, thereby enhancing the persuasiveness of our conclusions.

**Conclusion:**

About the question “This section effectively summarizes the significance of the results and their implications for the literature and clinical practice. However, the limitations of the results should have been discussed at the end of the discussion section rather than in this section.”

**Reply:** Thank you very much for your recognition and professional guidance on this section. At the same time, your suggestion that “the limitations of the results should have been discussed at the end of the discussion section rather than in this section.” precisely identifies an area for optimizing the content structure. We have adjusted the structure of the manuscript as per your guidance, moving the limitations mentioned in this section in full to the discussion section, and supplemented with specific analyses of the limitations and prospects for future research directions, ensuring that the content of each section is more focused and the logic is more coherent (Page 30, lines 691-700, and page 31, lines 701-702).

We sincerely appreciate your high recognition and professional evaluation of this research. This not only enables us to deeply appreciate your profound understanding of the research value, but also greatly boosts the confidence of the team in continuously exploring the field of traditional Chinese medicine modernization. We have further refined the manuscript's details to more fully convey the value of our findings and provide clinicians with more meaningful research support. Thank you once again for your thorough review and valuable encouragement.

**Reviewer #5:**

Thank you very much for your precise summary and professional recognition of the core mechanisms of this study. Your comment that “The paper presents a comprehensive mechanism where *Periplaneta americana* extract (PAD) treats ROUs not just locally, but by improving gut health. It regulates the gut microbiota, increases beneficial metabolites and strengthens the intestinal barrier which in turn reduces systemic inflammation that contributes to the formation and recurrence of oral ulcers.” perfectly aligns with the core of our research design and demonstrates your profound understanding of the mechanisms explored in this study. Once again, we would like to express our gratitude for your meticulous review and profound insights. Your professional feedback has provided us with crucial guidance in improving the manuscript and conveying the core value of the research. Next, we will respond to your questions and professional comments in the order you raised them.

1. About the question “Discussion on the correlation between oral and gut microbiota.”

**Reply:** Thank you very much for highlighting the important direction of “the association between the oral microbiome and the gut microbiome”. We have supplemented the relevant content in the Discussion section (Page 26, lines 584-593). Once again, we appreciate your professional advice. Your insights have helped us refine the discussion of our research mechanisms and enhance the academic integrity of the manuscript.

2. About the question “The study does not identify or isolate the bioactive compound responsible for the action which makes it difficult to standardize for therapeutic application or even just for laboratory replication.”

**Reply:** Thank you very much for your interest in PAD bioactive components and standardization issues. Your feedback provides valuable guidance for us to more clearly convey the research background and value. We have supplemented the discussion section with 40 PAD (*Periplaneta americana* extract) components previously identified by the research team through UPLC-Q-TOF-MS analysis. These include 7 amino acids, 7 alkaloids, 6 fatty acids, 5 nucleosides, 4 peptides, 3 sugars, and 8 other compounds (Page 23, lines 510-512). Additionally, formulations primarily composed of PAD (Kangfuxin Liquid) have been applied in the treatment of oral mucosal epithelial damage caused by radiation therapy for tumors. The core objective of this study is to address the current situation where its clinical efficacy is well-established, but its mechanism of action remains unclear. We aim to delve into the specific pathways through which it improves oral ulcers by regulating the GM, thereby filling the gap in mechanism research. Thank you once again for your meticulous review and professional feedback, which has enabled the manuscript to more fully convey its value to PAD-related research.

3. About the question “The results show a decrease in the Lactobacilli concentration. This is counterintuitive as Lactobacillus is generally a probiotic bacterium beneficial for gut health. The discussion does not discuss why the treatment would lead to a reduction in a beneficial genus that is abundant in the control group.”

**Reply:** Thank you very much for raising this profound and crucial question. The “beneficial nature” of Lactobacillus as a classic probiotic is not absolute; changes in its abundance must be comprehensively analyzed in conjunction with specific pathological microenvironments, therapeutic intervention mechanisms, and microbial community interactions. The gut is a complex ecosystem where each bacterial genus occupies a specific ecological niche. When a particular genus declines, its niche may be filled by other genera with similar functions. We have added a dedicated analysis in the “Discussion” section, incorporating the latest research findings in the field to provide the following supplementary explanation of the potential mechanisms underlying this phenomenon: Notably, following PAD intervention in rats with ROU, the abundance of probiotic Lactobacillus decreased. Although the total Lactobacillus count declined, the proliferation of Bifidobacterium helped maintain intestinal short-chain fatty acid levels. This suggests that functional redundancy within the gut microbiota may have offset the effects of reduced abundance in specific bacterial genera. Therefore, the core objective of PAD therapy for ROU is to break the “ROU-related gut microbiota dysbiosis cycle,” rather than simply modulating the abundance of single beneficial bacteria such as Lactobacillus (Page 26, lines 596-601, page 27, lines 602-626, and page 28, lines 627-629).

4. About the question “The paper provides compelling evidence for PAD’s efficacy through gut-oral axis, its primary limitation is the lack of oral microbiota data, uncharacterized crude extract and the lack of clarity whether the improved intestinal barrier is a direct result of PAD’s action or an indirect consequence of the modulated gut microbiota and increased SCFA production.”

**Reply:** We sincerely appreciate your high recognition and precise, insightful feedback. We have incorporated the limitations you identified in this study into the Discussion section (Page 30, lines 691-700 and page 30, lines 701-702). Your feedback objectively acknowledges the study's shortcomings while providing a well-reasoned analysis grounded in field advancements and experimental logic, along with a clear plan for subsequent validation. We sincerely appreciate your professional input once again. Your suggestions have helped us pinpoint the core gaps in our mechanistic research and chart a clear path for advancing our future investigations.

5. About the question “The discussion section is more like a combined version of the introduction and the results. The author has not discussed the results with other available research on ROUs or PAD as such. The discussion part could be rewritten to give a persuasive validation for the hypothesis made in the study.”

**Reply:** We sincerely appreciate your key suggestions. Your suggestions precisely pinpointed the core areas for improvement in the discussion section, enabling us to successfully restructure the argumentation logic and significantly enhance the depth and rigor of the content. We have thoroughly optimized the structure and core content of the discussion section in strict accordance with the journal's requirements and your guidance. We extend our sincere gratitude once again—your professional feedback has provided crucial support in enhancing the manuscript's quality.

6. About the question “The study demonstrates PAD’s effectiveness over a 7-day treatment period. A key feature of ROUs is their recurrence. While the introduction notes that Traditional Chinese medicine can inhibit recurrence rates, the short duration of the study does not allow for long term effects on preventing ROUs. Since the study is based on the hypothesis of amelioration of ROUs by enhancing gut microbiome, the sustainability of the changes in gut microbiota and intestinal barrier should be made clear. Effective arguments regarding the sustainability of the same after the treatment dosage reduction or cessation should also be mentioned to strengthen the correlations made in the study.”

**Reply:** Thank you very much for your highly targeted and constructive suggestions. You have accurately identified key directions for our research regarding long-term efficacy validation and the exploration of sustainable mechanisms, which provides a clear research path for our subsequent in-depth studies. The 7-day treatment period design in this study primarily references the conventional treatment duration for the acute phase of ROU in clinical practice. In clinical settings, the natural healing cycle for ulcers in ROU patients ranges from 7 to 10 days. Seven days represents a critical time point for evaluating ulcer healing and inflammation resolution. Therefore, this study focuses on this cycle, with the core objective being to validate the immediate efficacy of PAD in improving acute-phase symptoms of ROU.

[1] Abbasi F, Raoof M, Khatami R, Shadman N, Borjian-Boroojeni F, Nazari F. Effectiveness of Amlexanox and Adcortyl for the treatment of recurrent aphthous ulcers. J Clin Exp Dent. 2016 Oct 1;8(4):e368-e372.

[2] Yuqiong W ,An’an S ,Xinran J , et al. Multidrug dissolvable microneedle patch for the treatment of recurrent oral ulcer. Bio-Design and Manufacturing, 2022, 6 (3): 255-267.

**Queries:**

1. About the question “Does the Kangfuxin liquid also work through a similar mechanism in reducing the ROUs as its raw material is also PAD and has been in use for a long time?”

**Reply:** Thank you for your valuable questions. The core active ingredient source of Kangfuxin Liquid is its raw material PAD. This study has demonstrated that PAD can achieve the therapeutic effect on ROU by regulating the intestinal microbiota, increasing beneficial metabolites, and enhancing the intestinal barrier, thereby reducing systemic inflammation. This provides a fundamental basis for the correlation of the two mechanisms of action. Based on the proven therapeutic effect of ROU in the long-term clinical application of Kangfuxin Liquid, it is reasonable to speculate on the core pathway through which it functions. It is highly likely to be related to the mechanism of action of PAD revealed in this study. At the same time, it is necessary to objectively state that besides PAD, Kangfuxin Liquid also contains auxiliary ingredients. These ingredients may have subtle adjustments on the action process. Therefore, further experimental verification is still required in the future.

2. About the question “Mention the primer sequence for the internal control for the qPCR and cite a reference for the same. Also cite a reference for the RQ calculation.”

**Reply:** We sincerely appreciate the detailed suggestions you have provided. These suggestions have offered crucial references for us to correct our shortcomings. We have supplemented the primer sequences for the internal control in the PCR in the “2.10 Reverse transcription quantitative PCR (RT-qPCR) analysis” section of the manuscript, along with the references for these sequences and the relevant references for RQ calculation (Page 12, lines 255-257).

3. About the question “IL-2 is seen in higher level in RAS patients and contributes to the release of TNF-α and IL-6. Why is the serum level higher in control and lower in the model group? (Section3.3, Fig. 1B).”

**Reply:** Thank you very much to the reviewer for raising this crucial question. After reviewing the literature, interleukin - 2 (IL-2) has been found to be involved in the pathogenesis of RAS. IL-2 is a key immunoregulatory factor mainly produced by CD4⁺ helper T cells of type 1 (Th1 cells). It plays a central role in the initiation, regulation, and effect exertion of the immune response in the body. IL-2 specific functions include promoting the proliferation and activation of T lymphocytes, inducing B lymphocytes to secrete specific antibodies, and enhancing the killing activity of natural killer (NK) cells. It is an important molecule maintaining the balance between cellular immunity and humoral immunity. The results of this study show that the serum IL-2 level in the RAS model group of rats was significantly lower than that in the control group. The possible mechanism for this is as follows: The number of Th cells in the peripheral blood of the model rats decreased, directly leading to a decline in the synthesis and secretion capacity of IL-2; and the deficiency of IL-2 would further cause impaired cellular immune function, weakening the body's ability to clear local inflammation and repair oral mucosal injuries, ultimately inducing pathological damage similar to human RAS (Page 25, lines 559-565).

4. About the question “Section 3.3, why IL-2 expression not determined among all the other cytokines expressed in serum? (Fig. 1C).”

**Reply:** Thank you for your detailed inquiry and professional consideration. We have supplemented our analysis with PCR technology to examine IL-2 mRNA expression levels in rat oral mucosal tissue. The results indicate that following PAD intervention, IL-2 mRNA expression levels were significantly upregulated in a dose-dependent manner. This trend aligns with the expression characteristics of IL-2 in rat serum (Page 15, line 321 and Fig. 1C).

5. About the question “Section 3.11 & Fig 8A, SIgA significantly reduced in the model group seems not to be the right term as compared to the treatment group, the levels of SIgA are on a similar level.”

**Reply:** We sincerely apologize for any inconvenience caused by our previous wording. We have revised the expression in Section 3.11 to read “The model group had significantly decreased SIgA content in the colon tissue than the control group. Compared with the model group, all treatment groups showed an upward trend in SIgA levels in colonic tissue, but this increase was statistically significant only in the PAD-H and LM groups (Fig 8A).” (Page 21, lines 454-458).

6. About the question “Levamisole hydrochloride (LM), has been used as positive control in the study. Since LM is commonly used in clinical treatments of ROUs, mentioning its side effects or why PAD could be better than commercially available drugs could be included in either the introduction or the discussion section- justification required.”

**Reply:** Thank you very much for your constructive suggestions. We have promptly reviewed the information regarding LM side effects and supplemented the discussion section accordingly (Page 24, lines 539-541). The following is added: However, during the treatment of ROU with LM, patients may experience side effects such as gastrointestinal reactions and neurological reactions, with an incidence rate of 11.2%.

**Major corrections**

1. About the question “Periplaneta americana extract in abbreviated (PAD) form has not been mentioned prior to Line 71.”

**Reply:** Thank you very much for your thorough review and reminder. We provided the full definition of the abbreviation “PAD” when it first appeared in the “Introduction” and have reviewed the use of abbreviated terms throughout the text (Page 5, lines 80-81).

2. About the question “Line 100: Butyric has been mentioned twice.”

**Reply:** Thank you very much for your meticulous and rigorous review. After verification, in the original manuscript, the original text on line 100 contained a redundant expression “Butyric”. We have deleted one of the duplicate contents and corrected the sentence (Page 6, line 111). The revised sentence ensures that the name appears only once, accurately conveying the experimental material information and also meeting the concise and standardized expression requirements of academic manuscripts, avoiding any interference with the readers' understanding.

3. About the question “Lines 117-124 could be written as a sentence. Mentioning each instrument separately makes it incomplete.”

**Reply:** We sincerely thank you for pointing out the deficiencies in the information presented in the “2.1 Materials and reagents” section. We have made adjustments to the relevant content: We removed the part “UPLC-Q-TOF-MS (Agilent, USA)…SPSS 26.0 statistical software (SPSS Inc., Chicago, IL, USA)” from the original manuscript from the “2.1 Materials and reagents” section, and then separately added it to the experimental steps in the "Methods" section to make the information more logically organized (such as page 6, line 127, page 7, lines 128-134, page 10, lines 201, 213, and page 11, lines 232, 234-235, 247-248).

4. About the question “Line 127: medicinal material has been mentioned twice. Suggestion: “Briefly, 540g of Periplaneta americana (medicinal material) was crushed into a powder using a mortar and pestle.” (or whatever instrument was used to grind the insects)”

**Reply:** Thank you very much for your thorough review and specific suggestions. You pointed out that the term “medicinal material” was repeated in line 127 of the original manuscript. This feedback effectively helped us address redundancy issues in the manuscript, and we promptly made the suggested revisions. We have corrected it to “Briefly, 540g of *Periplaneta americana* (medicinal material) was crushed into a powder using a herbal pulverizer” (Page 6, lines 137-139).

5. About the question “Section 2.3: The title of the section sounds vague.Suggestions: Experimental model, research model or model organism.”

**Reply:** Thank you very much for your precise suggestions. However, this section primarily focuses on the rearing conditions and appropriateness of the experimental animals used in this study. Therefore, we recommend revising “2.3 Animals” to “2.3 Experimental animals” (Page 7, line 146). This new title accurately conveys the core content of the chapter. Compared to the original title, it better adheres to the academic writing standard of "highly matching chapter titles and contents", and also helps readers quickly grasp the core information of this section.

6. About the question “Mention the diet for the model organism used in the study.”

**Reply:** Thank you very much for your valuable suggestions. You have pointed out the need to supplement the dietary information of the experimental organisms in the study. This detail will further enhance the completeness and reproducibility of the experimental methodology. We have promptly verified and supplemented the relevant information. We have added “They were allowed to freely access food and water.” to section “2.3 Experimental model” (Page 8, lines 152-153).

7. About the question “The term model group can be misinterpreted. Standard term “negative control” can be used to indicate rats administered with saline solution.”

**Reply:** Thank you very much for your valuable suggestions. The naming logic of “model group” in the manuscript is that mice simultaneously receive normal saline solution and antigen emulsifier, aiming to establish an experimental model. While the “negative control group” you mentioned has a standard definition of only receiving normal saline treatment without including the key experimental intervention factor. In this manuscript, it has already been named as “control group”.

8. About the question “Fig 6A: Change “gobler cell” to “goblet cells”.”

**Reply:** Thank you very much for your detailed correction. We have confirmed that there is a spelling error in Figure 6A, where "gobler cell" is a mistake and has been corrected to the correct term "goblet cells" to ensure the accuracy and consistency of the professional terminology (Fig. 6A). Once again, we thank you for helping to enhance the rigor of the details of the manuscript.

9. About the question “Fig legend: either mention ROUs as in Fig 1, 5, 6 or recurrent oral ulcers as mentioned in Fig 3, 4.”.

**Reply:** Thank you very much for your careful suggestions. We have noticed that there are two expressions, “ROUs” and “recurrent oral ulcers”, in the Fig legend. To ensure consistency in terminology throughout the entire document, we have standardized all the relevant descriptions in the legend and the main text as “ROUs” (Page 33, lines 765, 768, and 770). Once again, we sincerely appreciate your assistance in enhancing the meticulousness of the details of this manuscript.

10. About the question “Use the abbreviated form for an organism’s name throughout the manuscript after its first mention. (ex. Discussion line 477: *Periplaneta americana* to *P. americana*)”.

**Reply:** Thank you very much for your professional advice. When “*Periplaneta americana*” is first mentioned in the entire text, its Latin scientific name should be marked. In the subsequent content, the abbreviation “*P. americana*” should be uniformly used (Page 23, line 519). At the same time, all biological names in the paper have been thoroughly checked to ensure that after the first mention, they all adopt the standard abbreviation form, which conforms to the academic expression norms.

**Minor corrections:**

1. About the question “Follow the same tense through the manuscript (ex. Line 193: sulfuric acid was added and then in the same paragraph Line 195: microporous surface is used to)”.

**Reply:** Thank you very much for your thoughtful reminder. Your suggestion to maintain consistent tense usage throughout the manuscript is crucial for enhancing the document's standardization and readability. We have immediately initiated a comprehensive review and adjustment of tense usage across the entire text (Page 10, lines 212-213). We sincerely appreciate your professional feedback once again; your guidance has helped us avoid minor expression issues and elevate the manuscript's academic rigor.

2. About the question “Graphical abstract caption “ameliorates effect” to “ameliorating effects”.”

**Reply:** Thank you very much for your meticulous review and precise corrections. We have changed “ameliorates effect” in “Graphical abstract caption” to “amelioratory effects”. This adjustment corrects the grammatical expression and ensures the accuracy of the English wording. We have completed the modification immediately (Page 3, line 48).

3. About the question “Line 93: “material and methods” to materials and methods”.”

**Reply:** Thank you very much for your meticulous and rigorous review. Your careful attention has helped us further standardize the manuscript's formatting details. We have revised “material and methods” on line 93 of the original manuscript to “materials and methods” (Page 6, line 103).

4. About the question “Line 414: “antigen emulsifier” to “Antigen emulsifier” (Sentence case)”.

**Reply:** Thank you very much for your detailed corrections. We have revised the original manuscript's “antigen emulsifier” to “Antigen emulsifier” (Page 20, line 439).

5. About the question “Fig 1A legend: Line 635 ‘theoral mucosa’ to ‘the oral mucosa’”.

**Reply:** Thank you very much for your careful review of the manuscript. We have revised the original manuscript's “theoral mucosa” to “the oral mucosa” (Page 33, line 753).

6. About the question “Fig 6 legend: Line 661 add spacing”.

**Reply:** Thank you very much for your comments. We have promptly corrected the original manuscript's “theoral mucosa” to “the oral mucosa” and reviewed the entire text to ensure no similar spelling or formatting errors remain (Page 34, lines 778-782).

**Reviewer #6:**

**Major Comments**

1. About the question “Model justification: The antigen emulsifier-induced ROU model is not standard. The authors should provide stronger justification that this model reflects the pathophysiology of human recurrent aphthous ulcers, and discuss its limitations”.

**Reply:** Thank you very much for this central question. You have pointed out the need to further provide evidence supporting the “validity of the antigen emulsifier-induced ROU model” and discuss its limitations. This requirement directly relates to the clinical translational value of the research findings. We have promptly reviewed the evidence demonstrating the pathophysiological correspondence between this model and human ROU and supplemented the systematic analysis in the Discussion section (Page 22, lines 485-489, page 22, lines 493-501, and page 23, lines 502, 505-507).

2. About the question “The conclusion should be tempered to emphasize association rather than causality”.

**Reply:** We sincerely appreciate the reviewers' valuable suggestions. We have systematically reviewed and revised each sentence in the conclusion section, rigorously adhering to the core requirement of emphasizing correlation rather than causation to optimize the wording (such as page 24, lines 542-551, page 25, lines 553-570, 573-576, and page 24, lines 577-580). Once again, thank you for your professional feedback, which has provided crucial support in enhancing the manuscript's academic rigor.

3. About the question “Overstated conclusions: The manuscript at times suggests PAD is an “effective treatment” for ROUs. Given that the data are preclinical (rat model only), the discussion should highlight translational limitations and avoid clinical overstatements.”.

**Reply:** We sincerely appreciate the reviewer's valuable comments, which accurately identified the inadequacies in the manuscript's conclusions. We have comprehensively revised all statements extending beyond the preclinical scope of this study (Page 31, lines 704-723) and specifically supplemented the discussion section with an analysis of the study's limitations (Page 30, lines 691-700 and page 31, lines 701-702). This ensures that the presentation is rigorously aligned with the actual stage of the research.

4. About the question “Clarity and redundancy: The Introduction and Discussion are long and at times repetitive. Streamlining would improve readability.”.

**Reply:** Thank you very much for your valuable suggestions. We have immediately undertaken a systematic streamlining and optimization of these two chapters. By consolidating redundant content and focusing on core logic, we have significantly enhanced the readability and professionalism of the manuscript. Once again, we appreciate your expert input.

**Minor Comments**

1. About the question “Figures and legends should be checked for clarity (some are difficult to interpret in the PDF).”.

**Reply:** Thank you very much for your important reminder. We have immediately initiated a comprehensive review and optimization of all figures throughout the manuscript to ensure that the clarity of every figure meets publication standards.

2. About the question “Abbreviations (PAD, ROU, SCFA, GM) should be redefined in Results for reader convenience.”.

**Reply:** Thank you very much for your practical suggestions. Considering that the "Results" section is the core chapter where readers focus on the experimental data and conclusions, and there may be cross-chapter reading, we have redefined each abbreviation when it first appears in this section (such as page 14, lines 290-291, page 15, lines 315-316, 322, and page 16, lines 328-329).

3. About the question “Ethical approval is mentioned, but animal welfare descriptions could be expanded (analgesia, humane endpoints).”.

**Reply:** Thank you very much for your important suggestions. We have added details regarding animal welfare to the “2.5 Preparation of biological samples” section (Page 9, lines 185-189). As follows: After 24 h of fasting, the rats were humanely euthanized by intraperitoneal injection of sodium phenobarbital. Throughout the research, all animals remained in good health, and no unexpected deaths occurred. The rats were subsequently disposed of in accordance with the guidelines set forth by the National Research Center’s Safety and Health Committee (NRC).

4. About the question “The limitation section briefly mentions oral flora, but further elaboration is needed (e.g., small sample size, lack of female animals).”.

**Reply:** Thank you very much for your constructive suggestions. The supplementary content has now been integrated into the “Discussion” section (Page 30, lines 691-700 and page 30, lines 701-702), ensuring logical coherence and detailed specificity in the limitations analysis while providing clear direction for future research. We sincerely appreciate your professional feedback once again; your recommendations have significantly enhanced the depth and rigor of our discussion.

5. About the question “References: Some claims in Introduction and Discussion would benefit from more recent citations.”.

**Reply:** Thank you very much for your valuable suggestions. You pointed out that certain assertions in the Introduction and Discussion sections require additional support from recent literature. This adjustment will significantly enhance the timeliness and persuasiveness of our research arguments. We have immediately commenced work on supplementing the literature and optimizing citations. Once again, we appreciate your professional feedback; your suggestions have helped us strengthen the literature foundation of our research.

6. About the question “Grammar and syntax require polishing for international readability.”.

**Reply:** Thank you very much for your professional advice. Currently, we have completed the polishing of the entire manuscript, ensuring that the language expression conforms to the norms of international academic writing.

We are very thankful to you for allowing us to resubmit the revised version of our manuscript.

All authors have revised and approved the new version of the manuscript. We hope that the revised manuscript is now deemed suitable for publication in your esteemed journal, the PLOS ONE.

Sincerely,

Peiyun Xiao

Provincial Department of Education, Dali University, Dali 671000, China

No. 22 Wanhua Road, Dali, Yunnan 671000, China

Tel.: +86-15808729786

Fax: +86-0872-2256269

E-mail: xiaopeiyun@dali.edu.cn
