# Supplementary material for: Periplaneta americana extract ameliorates recurrent oral ulcers in rats by enhancing the intestinal epithelial barrier and regulating gut microbiota
Source: PLoS One. 2026 Jan 23;21(1):e0340453. doi: 10.1371/journal.pone.0340453 (PMC12829797; doi:10.1371/journal.pone.0340453)
Supplement: Attachment — Submitted filename: Decision on Manuscript IDPONE-D-25-27151R1.docx [file pone.0340453.s005.docx]

**Decision on** **Manuscript ID: PONE-D-25-27151**

**Title: *Periplaneta americana* extract ameliorates recurrent oral ulcers in rats by enhancing the intestinal epithelial barrier and regulating gut microbiota**

After thoroughly evaluating the revised manuscript and cross-checking all changes against the original reviewer comments, I find that the authors have fully addressed all concerns raised during the previous round of review. The manuscript has improved substantially in scientific clarity, structural coherence, and methodological transparency. I recommend acceptance of the manuscript in its current revised form.

**Comments:**

The authors have submitted a carefully revised version of the manuscript demonstrating meaningful improvements across conceptual, methodological, and presentation aspects. All major criticisms including model justification, moderation of causal claims, and reduction of clinical overstatement have been fully resolved. The manuscript is now scientifically sound, clearly written, and suitable for publication.

Below is a detailed assessment of how each concern has been addressed:

1. Justification of the ROU Model

The revised Introduction and Methods now provide an expanded rationale for using the antigen–emulsifier-induced ROU rat model. The authors have also clearly acknowledged the limitations of this model in the Discussion. This satisfactorily resolves the concern.

2. Moderation of Mechanistic and Causal Claims

The manuscript previously implied direct mechanistic causality between PAD treatment and gut-oral axis modulation. The authors have now appropriately adjusted the language to emphasize associations rather than causality, which is appropriate for a preclinical animal study.

3. Removal of Overstated Clinical Claims

Statements describing PAD as an “effective treatment” have been corrected. The revised text now correctly frames PAD as a therapeutic candidate requiring further investigation, which aligns with the scope of a preclinical model.

4. Improved Organization and Reduction of Redundancy

The Introduction and Discussion have been streamlined, reducing repetition and improving readability. The narrative flow is now much clearer.

5. Figure and Legend Clarity

All figure legends have been revised for better clarity and uniformity. Terminology, labeling, and descriptions are now consistent and comprehensible.

6. Clarification of Abbreviations

The authors have redefined all abbreviations (PAD, ROU, SCFA, GM) upon their first appearance in the Results, improving reader accessibility.

7. Ethical and Animal Welfare Descriptions

The revised ethical statement includes additional details regarding housing conditions, diet, analgesia, and humane endpoints, addressing concerns about animal welfare reporting.

8. Strengthened Limitations Section

The limitations now include sample size constraints, lack of female animals, limited oral flora assessment, and inherent model limitations. This addition significantly improves the manuscript’s transparency.

9. Updated and Relevant References

More recent references have been incorporated, particularly in the Introduction and Discussion, aligning the manuscript with current literature.

10. Language and Grammar Corrections

The manuscript has undergone evident language polishing. Issues related to grammar, sentence structure, and clarity have been addressed, resulting in a more professional and readable document.

11. Correction of Typographical Errors

All previously noted typographical errors (e.g., “gobler” → “goblet cells,” “theoral” → “oral”) have been corrected.

12. Improved Clinical Context

The Introduction now includes a clearer and more medically accurate description of recurrent oral ulcers and their clinical relevance.

13. Additional Information on PAD Composition

The authors have added GC–MS profiling of the PAD extract, including details on 40 identified chemical constituents. This adds depth to the methodological rigor.

14. Methodological Enhancements

Key reagent details, PCR conditions, dosing schedules, and procedural descriptions have been expanded in the Methods section. These revisions improve reproducibility.

15. Clarified Animal Housing and Diet

The authors have clearly stated the housing environment, diet type, temperature, and light cycle conditions.

**Overall Evaluation**

The authors have made substantial and appropriate revisions. The manuscript now presents:

A justified animal model

Balanced interpretation of findings

Improved methodological transparency

Clear and consistent figures

Enhanced readability

Stronger adherence to ethical reporting standards

The revised manuscript is scientifically sound and suitable for publication without further modification.

**Final Recommendation: Accept**
